# Controlling the Nematic Liquid Crystallinity of Cellulose Nanocrystals with an Alcohol Ethoxy Sulfonate Surfactant

**DOI:** 10.1021/acs.biomac.3c01375

**Published:** 2024-03-20

**Authors:** Johanna Majoinen, Lotta Gustavsson, Owies Wani, Samira Kiefer, Ville Liljeström, Orlando J. Rojas, Patrice Rannou, Olli Ikkala

**Affiliations:** † Department of Bioproducts and Biosystems, Aalto University, Aalto, FI-00076 Espoo, Finland; ‡ Technical Research Centre of Finland VTT, Biomaterial Processing and Products, FI-02150 Espoo, Finland; § Department of Applied Physics, Aalto University, Aalto, FI-00076 Espoo, Finland; ∥ Nanomicroscopy Center, OtaNano, Aalto University, Aalto, FI-00076 Espoo, Finland; ⊥ Bioproducts Institute, Department of Chemical and Biological Engineering, Department of Chemistry and Department of Wood Science, University of British Columbia, 2360 East Mall, Vancouver, British Columbia V6T 1Z3, Canada; # Université Grenoble Alpes, Université Savoie Mont-Blanc, CNRS, Grenoble INP, LEPMI, 38000 Grenoble, France

## Abstract

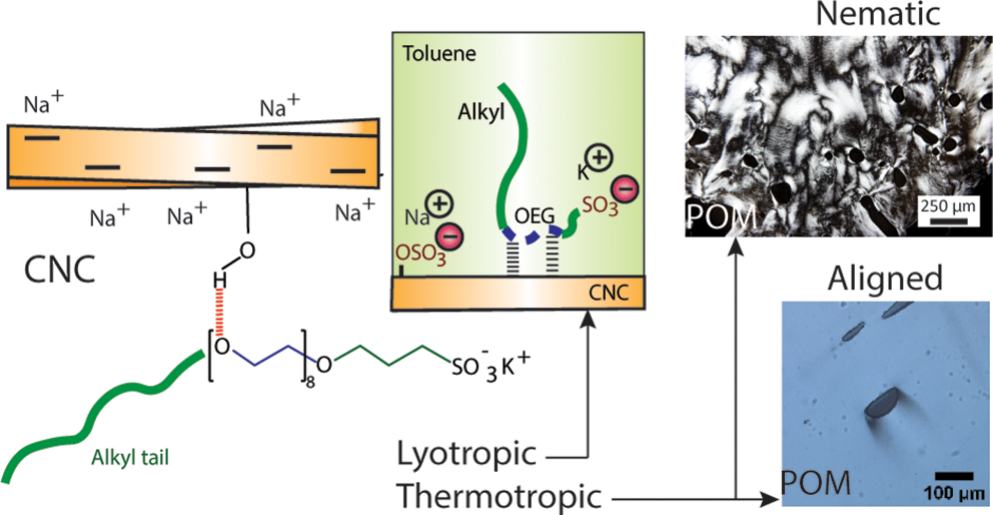

Cellulose nanocrystals (CNCs) are biobased colloidal nanorods that have unlocked new opportunities in the area of sustainable functional nanomaterials including structural films and coatings, biomedical devices, energy, sensing, and composite materials. While selective light reflection and sensing develop from the typical chiral nematic (cholesteric, Nem*) liquid crystallinity exhibited by CNCs, a wealth of technologies would benefit from a nematic liquid crystal (LC) with CNC uniaxial alignment. Therefore, this study answers the central question of whether surfactant complexation suppresses CNC chirality in favor of nematic lyotropic and thermotropic liquid crystallinity. Therein, we use a common surfactant having both nonionic and anionic blocks, namely, oligo(ethylene glycol) alkyl-3-sulfopropyl diether potassium salt (an alcohol ethoxy sulfonate (AES)). AES forms complexes with CNCs in toluene (a representative for nonpolar organic solvent) via hydrogen bonding with an AES’ oligo(ethylene glycol) block. A sufficiently high AES weight fraction endows the dispersibility of CNC in toluene. Lyotropic liquid crystallinity with Schlieren textures containing two- and four-point brush defects is observed in polarized optical microscopy (POM), along with the suppression of the cholesteric fingerprint textures. The results suggest a nematic (Nem) phase in toluene. Moreover, thermotropic liquid crystallinity is observed by incorporating an excess of AES, in the absence of an additional solvent and upon mild heating. The Schlieren textures suggest a nematic system that undergoes uniaxial alignment under mild shear. Importantly, replacing AES with a corresponding nonionic surfactant does not lead to liquid crystalline properties, suggesting electrostatic structural control of the charged end group of AES. Overall, we introduce a new avenue to suppress CNC chirality to achieve nematic structures, which resolves the long-sought uniaxial alignment of CNCs in filaments, composite materials, and optical devices.

## Introduction

Driven by the quest for sustainability, cellulosic structures have been pursued to achieve value-added materials, benefiting the mission of carbon neutrality along with improved performance in next-generation bioproducts.^1−9^ Therein, colloidal cellulose nanorods (cellulose nanocrystals, CNCs) have been within the focal point, given their self-assembly features.^1,5,9,10^ CNCs of high aspect ratios and intrinsic mechanical strength can be isolated and used in applications that benefit from CNC biocompatibility, low density, and tailorable surface chemistries. CNC chiral organizations allow light (Bragg) reflection in structural colors,^9,10^ which suggest application potential in photonics, tissue scaffolding, packaging, as well as stimuli-responsive, biomedical, and polymer-reinforced materials.^1,5,11−14^ Classically, CNCs form cholesteric lyotropic liquid crystal (LC) (Nem*) phases in aqueous suspensions, which are retained even in the dry state, giving rise to structural colors and infrared reflectors.^15−19^ To make CNCs dispersible in organic solvents, their surfaces have been modified using supramolecular or covalent approaches, as well as physical adsorption (surfactants, polymers, etc.). Still, the resulting colloidal systems often preserve the chiral nematic (cholesteric, Nem*) order.^9,10,20−22^ The inherent tendency toward the chiral Nem* mesophase of CNCs has been beneficial in many applications, but it has remained challenging to understand, control, or suppress. The latter is a central objective of this work.

It has been suggested that the chiral phases form due to the twisted geometry of individual CNCs,^23,24^ CNC bundling,^25^ or the asymmetric distribution of the negatively charged sulfate esters on CNC surfaces.^26^ Overall, the mechanism of CNC chirality remains a topic that is both scientifically intriguing and technologically relevant. However, a wealth of applications could benefit from uniaxially aligned nematic CNC organizations instead of chiral cholesteric twists. For instance, suppressing CNC chirality can be useful in the synthesis of composites, filaments, fibers, and optical polarizers. Typically, a high electric or magnetic field leads to an increased pitch of the cholesteric twisting but fails to achieve CNC alignment.^27−29^ Moreover, strong shearing may kinetically trap only temporarily the thermodynamically unstable aligned organization, eventually returning to chiral assemblies. Therefore, there is a need to find facile routes to prepare systems where CNC liquid crystal chirality is suppressed. In fact, only few mesophases other than chiral have been reported for CNCs, in which case, e.g., high viscosity/thixotropic gelling or added salts dictate the phase behavior.^22,23,30−34^

**Figure 1:**
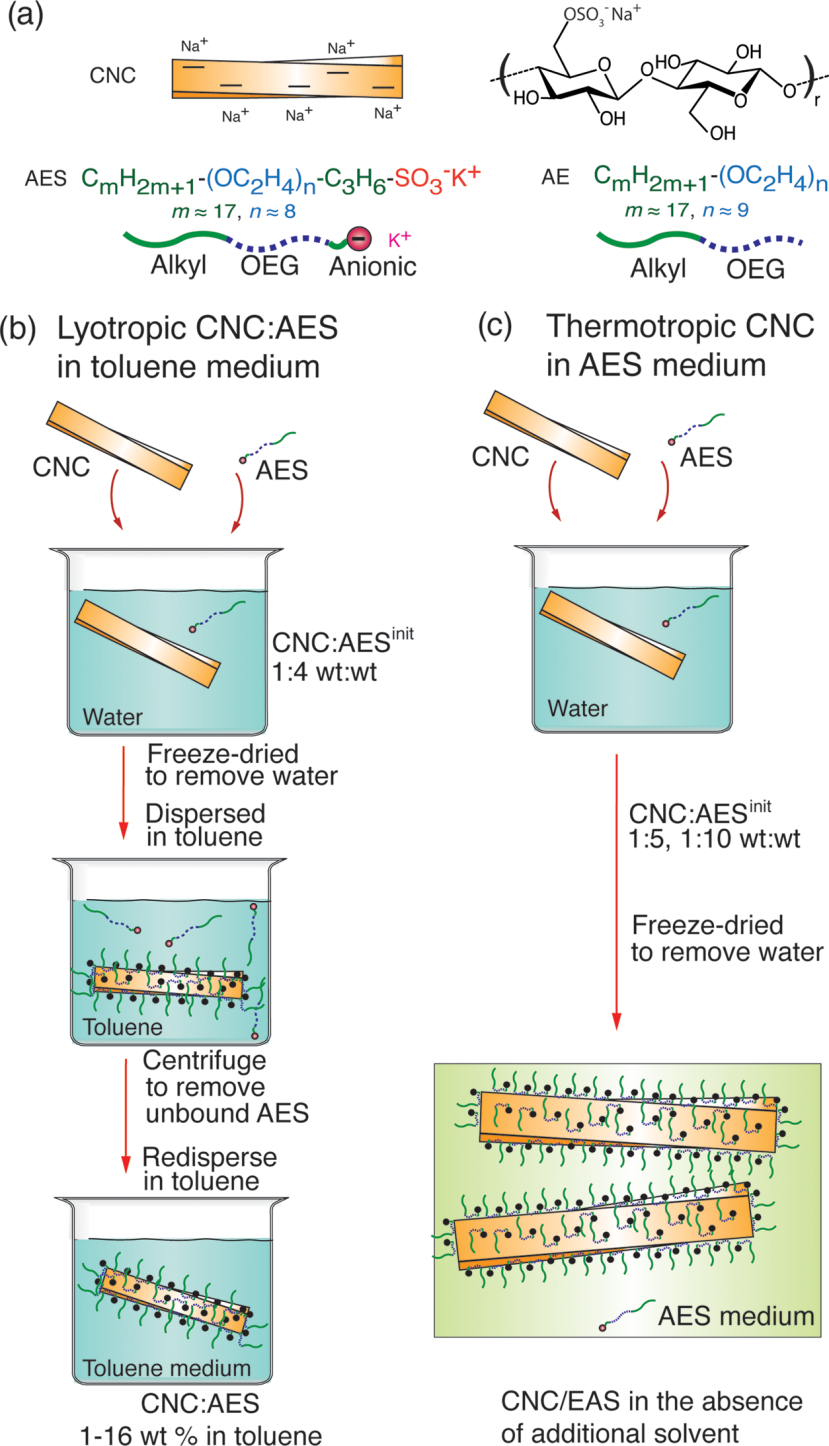
(a) Chemical structures of the cellulose nanocrystals (CNCs), the nonionic–anionic trifunctional oligo(ethylene glycol) alkyl-3-sulfopropyl diether potassium salt (AES) surfactant, and the nonionic difunctional reference surfactant ethoxylated alcohol (AE) without the anionic end group. (b) Schematics for CNC/AES processing into lyotropic suspensions. (c) For the (solvent-free) thermotropic CNC/AES system, CNCs were first mixed with an excess of AES surfactant in water, followed by water removal by freeze-drying. No additional organic solvents were used in this case.

Herein, we first report on a trifunctional nonionic–anionic surfactant (oligo(ethylene glycol) alkyl-3-sulfopropyl diether potassium salt), i.e., an alcohol ethoxy sulfonate, referred hereafter as alcohol ethoxy sulfonate (AES) (Figure 1a) that enables the formation of a stable colloidal dispersion of CNCs in a nonpolar organic solvent (toluene). Upon progressively increasing the AES concentration, the characteristic fingerprint textures observed in an aqueous (aq.) medium are first observed even in toluene, suggesting chirality. However, upon further increasing the AES concentration, the cholesteric fingerprint textures are suppressed and Schlieren textures (typical for nematics) emerge. This suggests that the chiral CNC pitch is chemically opened, in analogy to the effect of electric or magnetic fields but, in this case, by chemical effects.^29^ Strikingly, upon further increasing the relative AES fraction, no fingerprint textures are resolved; instead, two- and four-point brush defect-containing Schlieren patterns emerge, suggesting the formation of a nematic phase. Finally, a nematic thermotropic liquid crystal is observed at a sufficiently large AES fraction under solvent-free conditions.

## Experimental Section

### Materials

Cellulose nanocrystals (CNCs) were sourced from the Forest Products Laboratory (U.S. Forest Service) via the University of Maine Process Development Center and acquired in a never-dried sodium form. According to our characterization, the CNCs have the average length of ca. 200 nm and lateral dimension of ca. 11 nm (Figure S1). The surface of CNCs includes negatively charged sodium sulfate half-ester groups resulting from sulfuric acid hydrolysis, leading to an aqueous ζ-potential of −45 mV (Table S1).

The trifunctional nonionic–anionic surfactant (oligo(ethylene glycol) alkyl-3-sulfopropyl diether potassium salt, i.e., C*_m_*H_2*m*+1_-(OC_2_H_4_)*_n_*-C_3_H_6_–SO_3_^–^K^+^, subsequently denoted as AES) was acquired from Raschig GmbH (type F11–13) (Figure 1a). ^1^H and ^13^C NMR spectroscopic characterizations of AES lead to average values of *m* ≈ 17 for the alkyl chain block and *n* ≈ 8 for the oligoethylene glycol (OEG) middle block (Figure S2). AES consists of three subunits: (i) a nonpolar *n*-alkyl end-block providing solubility in nonpolar solvents, (ii) a short, polar nonionic OEG block in the center (which we later show to bond to CNCs by hydrogen bonding in nonaqueous medium), and (iii) a charged potassium sulfonate headgroup (which leads to promoted CNC surface charging). To explore the effect of the anionic charge in the surfactant, we also explored a corresponding nonionic surfactant as a reference, which does not contain the potassium sulfonate anionic group, i.e., ethoxylated alcohol surfactant C*_m_*H_2*m*+1_-(OC_2_H_4_)*_n_* (Brij C10, subsequently denoted as AE, see Figure 1a). AE was acquired from Sigma-Aldrich. Its chemical characterization by ^1^H and ^13^C NMR spectroscopies suggested *m* ≈ 17, *n* ≈ 9 (Figure S3). All materials were used as received.

### Characterization

Polarized optical microscopy (POM) was performed with an Olympus BX53 M microscope equipped with a DP74 camera or with a Leica DM4500/DM4 P/1090 microscope with Leica/Canon EOS 60D cameras. Dynamic light scattering (DLS) was measured with Malvern Zetasizer Nano ZS90 from different concentrations and pH using Milli-Q water and toluene as solvent at 25 °C. Transmission electron microscopy (TEM) was done with a JEM3200FSC field emission cryo-TEM. The samples were prepared by depositing pristine CNCs from water and CNC/AES complexes from toluene (0.05 wt %) onto a carbon-coated Cu grid. The dimensions of pristine CNC and CNC/AES complexes were acquired by using ImageJ software from TEM images. A Quartz Crystal Microbalance with Dissipation (QCM-D) (E4 instrument, Q-Sense AB) was used to monitor adsorption of AES and AE in toluene on thin CNC films that were fixed on SiO_2_ sensors. Toluene was used also as a washing solvent. All measurements were performed at 23 °C under a constant flow of 100 μL min^–1^.

## Results and Discussion

### CNC Dispersions in Toluene in the Presence of a Nonionic–Anionic AES Surfactant: Composition Optimization to Suppress the Nem* Mesophase

**Figure 2:**
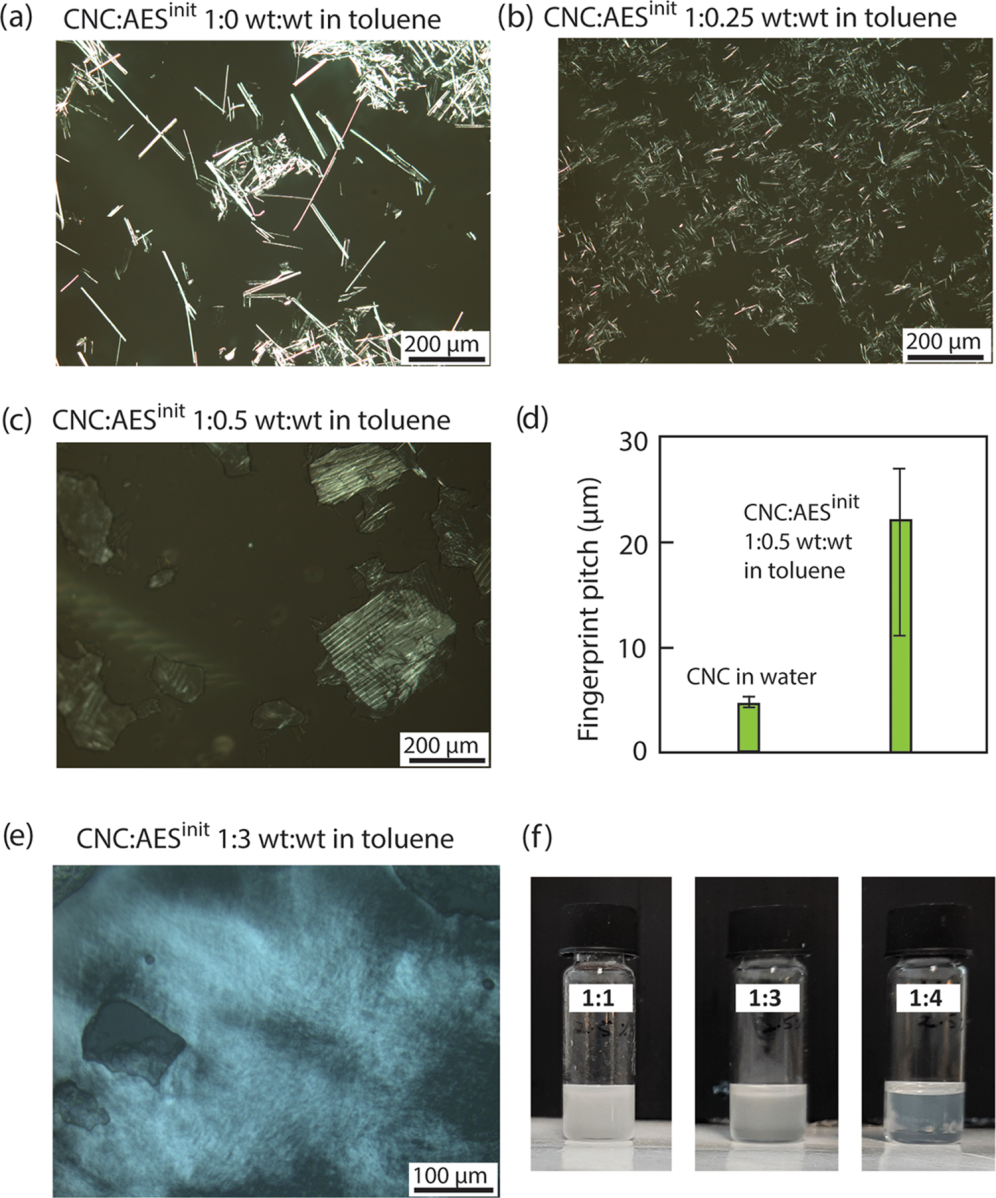
POM microphotographs (crossed polarizers) of CNC/AES^init^ compositions with their different weight fractions in toluene upon initial mixing in water, freeze-drying to remove water, and dispersion in toluene. (a) Pure CNCs show needle-like aggregates in toluene and (b) CNC/AES^init^ at low AES fraction 1:0.25 wt/wt shows softened edges. (c) At 1:0.5 wt/wt, soft aggregates with striped fingerprint patterns are observed in toluene, resembling Nem* self-assembly of the components but with considerably opened pitch in comparison to water (d). (e) CNC/AES^init^ 1:3 wt/wt in toluene showing birefringence but no fingerprint patterns. (f) Photographs of vials containing CNC/AES^init^ 1:1, 1:3, and 1:4 wt/wt in toluene.

CNC and the nonionic–anionic surfactant AES (Figure 1a) were first dispersed in water, corresponding to initial compositions CNC/AES^init^ 1:0, 1:0.1, 1:0.25, 1:0.5, 1:1, 1:3, and 1:4 wt/wt while keeping a fixed CNC aq. concentration of 1 wt %. The suspensions were subsequently freeze-dried to remove water and then redispersed in toluene at 50 mg/mL concentration (Figure 1b for schematics). POM was first used to qualitatively study the dispersions. Figures 2a–c and S4 present the POM images of CNC/AES^init^ dispersions in toluene at low AES relative fractions, ranging from CNC/AES^init^ 1:0 up to 1:0.5 wt/wt. Not surprisingly, the pristine CNCs (i.e., 1:0 wt/wt) severely aggregated in toluene, forming solid needle-like CNC crystals with sharp boundaries (Figure 2a). Upon slightly increasing the AES relative fraction, to CNC/AES^init^ 1:0.1 and 1:0.25 wt/wt, the needle-like aggregates appeared to have soft edges (Figure 2b and S4). However, at 1:0.5 wt/wt, a drastic change was observed where an assembly of CNCs to soft larger aggregates formed in toluene showing striped fingerprint patterns (Figure 2c), instead of poorly dispersed individual CNC rods. Notably, such patterns still suggest Nem* LC twisting, as was classically observed for CNCs in water. However, the fingerprint POM periodicity of CNC/AES^init^ 1:0.5 wt/wt in toluene was tens of micrometers, suggesting considerably larger cholesteric pitch compared to that in water, i.e., 4–5 μm (Figure 2d). The larger pitch suggests chemically induced opening of the chiral pitch in toluene induced by AES. Strikingly, related observations were made for aq. CNCs under strong magnetic or electric field, which produced an increased distance periodicity in the stripes, i.e., increased cholesteric pitch.^29^

This first hint already suggests that the nonionic–anionic AES surfactant affected the chirality of CNCs. Thus, one could ask whether further addition of AES allows for further increased cholesteric pitch, whereby an infinite value would asymptotically correspond to Nem LC, which is a desirable condition for uniaxial alignment. No cholesteric fingerprint patterns were observed after increasing the fraction of AES in CNC/AES^init^ to 1:3 wt/wt in toluene (Figure 2d). By contrast, large area birefringent domains were observed, indicating lyotropic LC behavior in toluene. A slight turbidity therein still indicated some residual aggregation (Figure 2e). Further increasing the fraction of AES, in CNC/AES^init^ 1:4 wt/wt, produced a system that was visually transparent, with no signs of aggregation, flocculation, or phase separation (Figure 2f). Thus, with the increased AES fraction relative to CNC, visually good birefringent dispersions in toluene were observed along with the suppression of chiral fingerprints formation.

Considering the results so far, two questions emerge: First, what is the interaction between the AES surfactant and CNCs? Taking into account that both CNCs and AES are anionic, electrostatic interactions are not expected. Second, what is the fraction of AES that is bound (complexed) onto CNCs?

To first explore the fraction of bound AES, the optically clear initial CNC/AES^init^ 1:4 wt/wt mixture in toluene was centrifuged to remove free AES. By weighing the separated dry fractions, it was found that the bound surfactant fraction corresponded to a CNC/AES mass ratio of ca. 1:1 wt/wt, next denoted as CNC/AES. Note that such a mass parity does not bear any obvious physical meaning. Such “washed” composition containing only bound surfactants was used in further experiments. The mechanisms of binding and complexation will be addressed later in this article.

### Surfactant-Bound CNC/AES Nem Lyotropic Mesophase in Toluene

**Figure 3:**
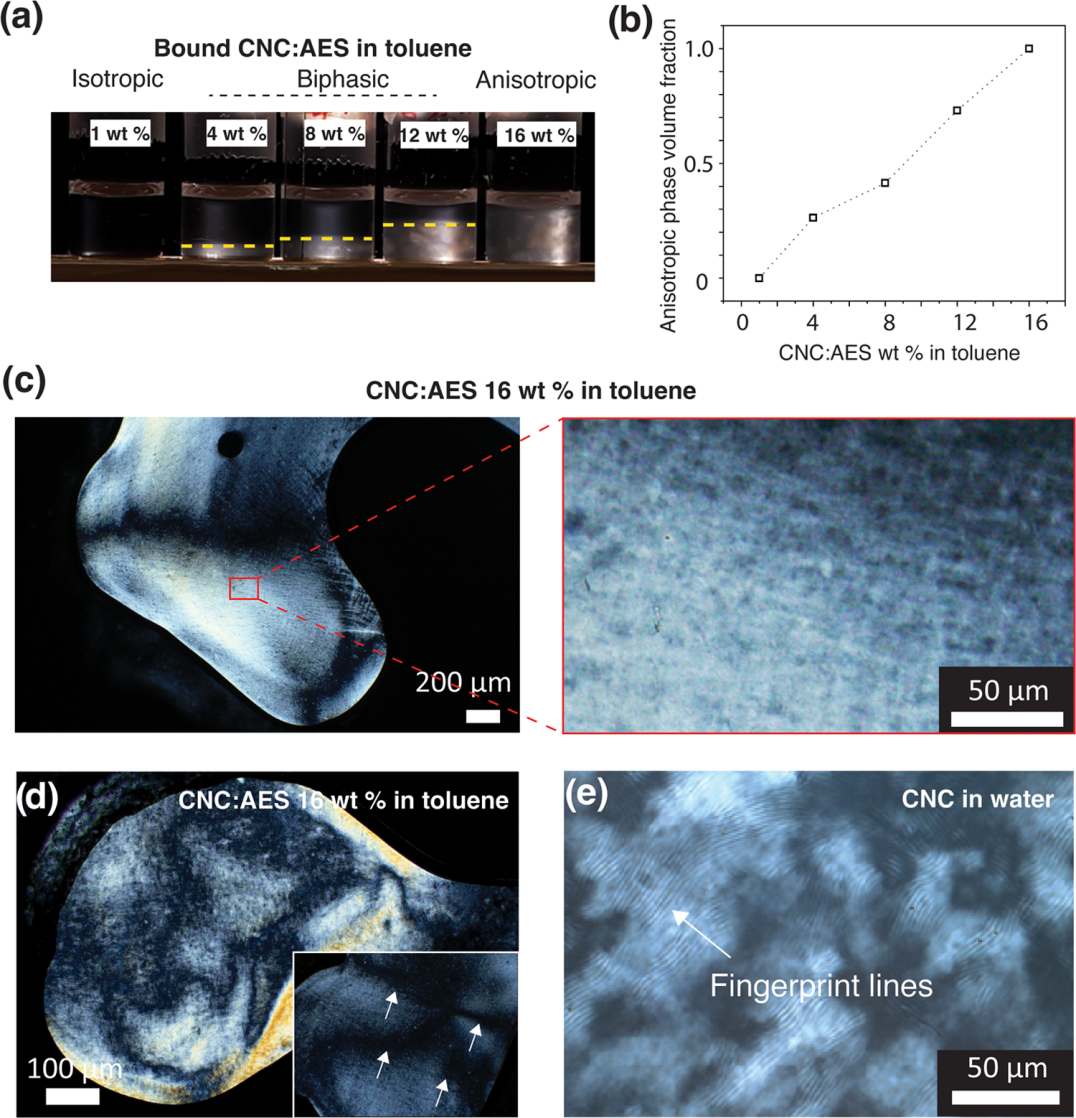
Liquid crystallinity of the “bound” CNC/AES complex dispersed in toluene. (a) Photograph of vials between crossed polarizers (with an angle of 90°) containing CNC/AES in toluene at increasing concentrations. The images were taken after 2 weeks of sample standing. The interface between the isotropic and the anisotropic phases is indicated by using yellow dotted lines. (b) Anisotropic phase volume fraction in toluene as a function of increasing complex concentration of CNC/AES (1–16 wt %). (c, d) POM microphotographs of CNC/AES complex 16 wt % dispersion in toluene captured from the Videos S1, S2 and S3 (Schlieren textures typical for Nem mesophase are observed, as seen as dark line features crossing the sample). The zoomed-in image in panel (c) shows the absence of any fingerprint texture. Inset in panel (d) shows a 4-brush (four white arrows) point singularity defect for CNC/AES complex 16 wt % in toluene. (e) As a control measurement, POM microphotograph of aq. CNC suspension with Nem* fingerprint lines.

Next, such washed CNC/AES compositions were dispersed in toluene at different concentrations. Figure 3a shows vials observed between crossed polarizers (i.e., 90° angle, unless otherwise stated) containing increasing concentrations of CNC/AES in toluene after equilibration for 2 weeks. Phase separation between an upper optically isotropic and lower optically anisotropic birefringent phase was observed, where the volume fraction of the latter phase increased approximately linearly as a function of added CNC/AES (Figure 3b). Upon reaching a CNC/AES concentration of ca. 16 wt % in toluene, the anisotropic phase roughly filled the whole volume.

Importantly, CNC/AES 16 wt % dispersion in toluene turned birefringent and highly fluid and, interestingly, did not show chiral fingerprints. By contrast, the sample showed highly dynamic Schlieren textures upon pressing the microscope coverslips (Video S1 for rotated Schlieren brushes and Videos S2 and S3 for pressed and rotated Schlieren brushes), which are clearly shown by POM universally assigned to Nem mesophase, also for colloidal rods.^35,36^ Selected images captured from the videos are shown in Figure 3c,d. Distinctly, four-brush point singularity (s = +1) Schlieren defects are identified in Figure 3d, a high magnification image captured from Video S2 as the polarizers were rotated anticlockwise and the patterns rotated in the same direction, strongly supporting a Nem mesophase. Further evidence for nematics (and to exclude the cholesteric classical structural color) is provided by the strong interference coloring and increasing birefringence of a POM sample with a pinned droplet when the Michel-Levy chart is utilized (Figure S5, not to be confused with structural colors). Coloring of the pinned droplet edge of CNC/AES in toluene appears due to anisotropic nanoparticles uniaxially aligned in a stack of tens of μm thickness, upon viewing this doubly refracting sample edge between crossed polarizers. The CNC/AES 16 wt % dispersion in toluene showed a pronounced flow birefringence upon shearing (see Videos S3 and S5 for dispersion fluidity and Video S4 for flow birefringence upon shaking a vial), suggesting facile alignment. The sample is a soft gel under quiescent conditions; however, with gentle shaking it became fluid-like, and it reformed a soft gel after standing overnight (Figure S6). We finally point out that in the LC-assemblies of CNC, kinetic trappings can take place^10^ and therein the presently observed low viscosity can be beneficial toward equilibrium structures.

**Figure 4:**
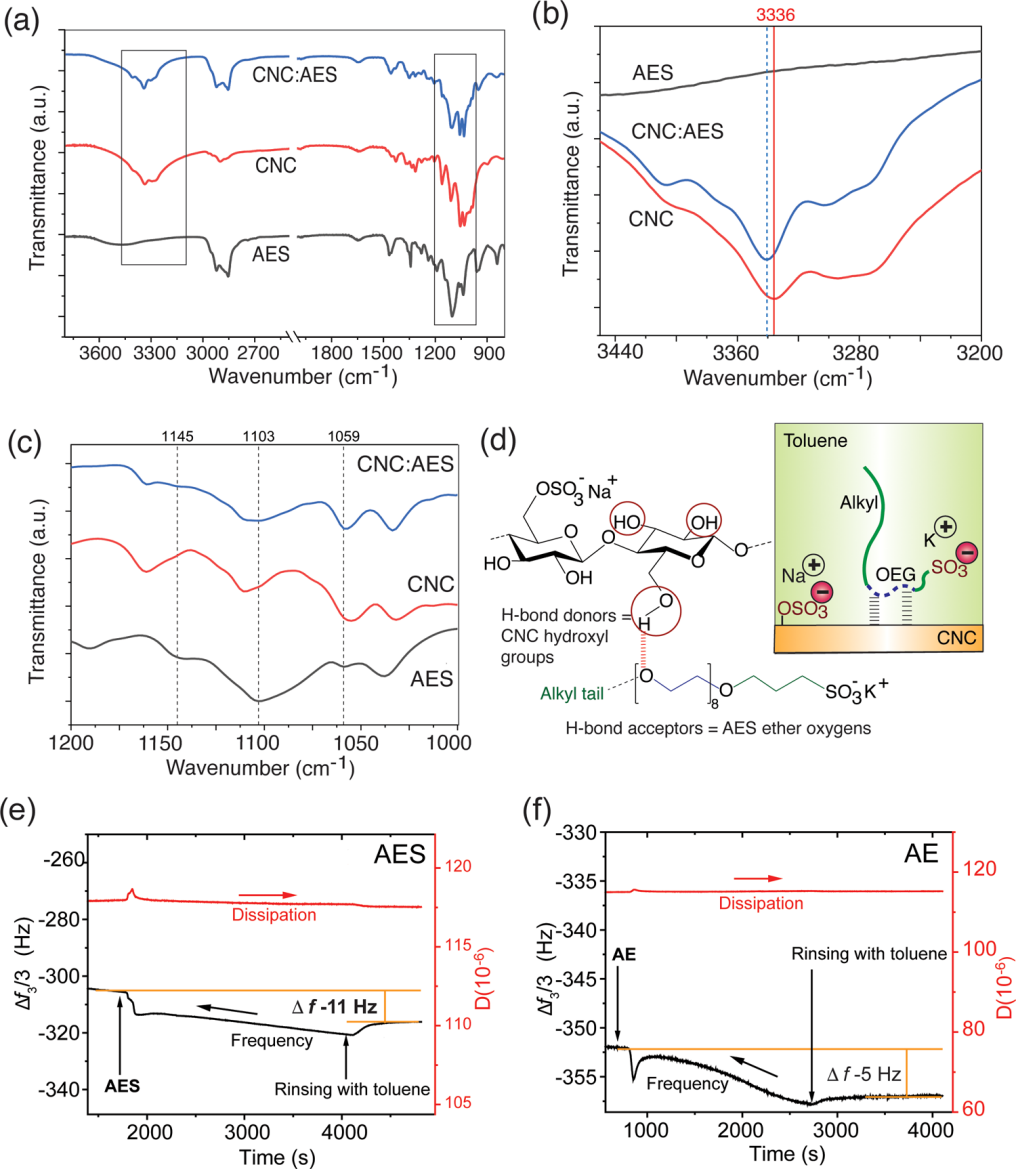
(a) FTIR spectra of pristine CNC, AES, and CNC/AES “bound” complex and zoomed in detail for the (b) OH stretching vibration of hydroxyl groups present onto the CNC surface and the (c) distortion of the three AES C–O–C stretching vibration IR bands (in black) in the complex spectra (in blue). (d) Proposed H-bonding interaction between CNCs and the OEG block of AES in the formed complex in toluene, thus providing high charge crowding on CNCs. QCM-D results of AES (e) and AE (nonionic reference surfactant) (f) adsorption on the CNC thin film surface. AES binds to the CNC surface according to Δ*f* −11 Hz frequency change after rinsing with toluene.

The interaction between CNCs and AES with the same charges is not trivial. In the aqueous medium, the AES surfactant does not electrostatically bind onto CNCs since both it and CNC are negatively charged and poly(ethylene glycol) is known to have no or only a weak binding with cellulose in water.^37^ However, the situation changes upon freeze-drying to remove water and dispersion in toluene. The present AES has a sufficiently long alkyl tail length to disperse CNC in toluene and thus to promote hydrophobic repulsion of the charged groups of AES from the nonpolar toluene. This obviously also has a strong effect on packing all charged groups near the surface of CNC. FTIR data of films cast from the bound CNC/AES in toluene suggest hydrogen bonding between the hydroxyl groups of CNCs (weak H-bonding donors) and ether oxygens of the OEG block in AES (H-bonding acceptors) (Figure 4a–d). The OH stretching band of CNC/AES between 3500 and 3000 cm^–1^ is narrow and shows a shift of ca. 5 cm^–1^ toward higher energy, compared to that of pristine CNCs (Figure 4b). The narrowing of the FTIR band indicates the reduction of intermolecular H-bonding between the neighboring CNCs and the shift in frequency indicates the development of H-bonding interactions between CNC and the OEG block of the AES.^28,38^ This is also evident from the analysis of a characteristic set of three IR bands present in AES, i.e., 1145, 1103, and 1059 cm^–1^, corresponding to C–O–C stretching vibrations. Peaks were distorted in CNC/AES spectra due to H-bonding interaction to CNCs (Figure 4c).^39^ Hence, it can be suggested that AES binds onto the CNC surface upon organic solvent processing step via H-bonding through the OEG functionality, and the nonpolar alkyl tail forms brushes on CNCs stabilizing the toluene suspension. The role of potassium sulfonate in AES is discussed later herein. Finally we also point out a potential subtlety: CNCs tend to incorporate a nanometric hydration layer despite the drying.^40^ Therefore, the observed hydrogen bonding and interaction can actually reflect an interplay of the hydrophobic interaction and hydrogen bonding.

To further shed light on these interactions, we selected a nonionic alcohol ethoxylated reference surfactant analogue AE (Figure 1a) as a reference in control experiments, having a structure similar to that of AES but with no potassium sulfonate group. CNC/AE^init^ 1:1, 1:2, and 1:3 wt/wt were prepared similarly as using AES, i.e., initially mixing in water, freeze-dried to remove water, and redispersing in toluene. Figure S7 shows the POM microphotographs of such CNC/AE^init^ mixtures with different compositions in toluene. AE did not allow a well-developed dispersion of CNCs in toluene nor LC phases, irrespective of the composition. Therefore, it is reasonable to conclude that the anionic group (potassium sulfonate end group of AES) also plays a subtle role in the initially observed phenomena.

Additional evidence of bonding between OEG block of AES and CNCs in the absence of water was provided by QCM-D binding experiments with AES and AE adsorbing on CNC thin films in toluene (Figure 4e,f) The frequency shift, Δ*f* = −11 Hz using AES after rinsing with toluene was larger than that measured for AE, Δ*f* = −5 Hz. The adsorbed mass of AES on CNCs after rinsing was 0.65 mg/m^2^ calculated from the Sauerbrey equation. Not unexpectedly, we did not observe the binding of AE on CNCs in toluene after rinsing (Figure 4f).

Therefore, both the nonionic OEG block and the anionic potassium sulfonate headgroup of AES play a role in the CNC dispersibility in toluene, binding, and formation of lyotropic LC. Based on the centrifugation experiments, designed to identify the fraction of AES that was bound to CNC, one can infer that there were ca. 3.2 AES molecules/nm^2^ adsorbed on CNCs (see the SI). Based on the Sauerbrey equation, the adsorbed AES amount is equivalent to 0.5 AES molecules/nm^2^ (SI). The approximated nature of these two methods does not allow quantitative agreement; still both suggest high AES packing density on CNCs. Therefore, an obvious effect of AES is to increase the original surface charge provided by –OSO_3_^–^Na^+^ of pristine CNCs by adsorbed −SO_3_^–^K^+^ mediated by hydrogen bonding with the AES surfactant, i.e., providing high packing of Na^+^ and K^+^ onto CNC. Note that the alkyl-nonionic surfactant AE, which does not include the anionic end group, provided no CNC dispersibility in toluene nor lyotropic LC behavior, thus directly pointing to charge effects.

The charging effects of colloids in nonpolar solvents are complex in general and not fully understood.^41,42^ It is known that surfactant-mediated charging can play a role in the stabilization of charged colloidal units even in low-polarity media,^43,44^ where the accumulation of charges at colloidal interfaces based on surfactants has been described as supercharging.^43^ On the other hand, adsorption of surfactants on CNCs leads to a change in the ζ-potential.^45^ Herein, compared to pristine CNC size and charges (in water), DLS measurement in toluene for the complexes showed a neutral value, and particle size was observed to grow (Table S1). This suggests that in toluene, the ionic moieties (including both anionic sulfonate as well as the sodium and potassium counterions) reside at the CNC surface, screening the negative surface potential of pure CNCs which enables the stable complex formation between CNC and AES, eventually leading to a nematic liquid crystal phase. We therefore suggest that the increased charging of CNCs is relevant, potentially related to CNC supercharging. Indeed, Araki and co-workers have reported a correlation between the proportion of sulfate groups on the surface of CNC obtained from microbial cellulose and the lyotropic LC behavior from Nem* to Nem due to changes in electrostatic interactions.^30^ We finally point out that complexation with cationic or anionic surfactants does not typically lead to the suppression of cholesteric twisting and even the dispersibility in organic media has proven to be challenging.^46^ Finally, triblock amphiphilic molecules, e.g., Pluronic polymers, have been shown to interact with nanoparticles for redispersion purposes.^47^ Previously, it was shown that CNCs can be dispersed in an organic solvent by using the ethoxylated phosphoric ester of a nonylphenol surfactant; however, the suppression of cholesteric twist has not been reported.^20,48,49^ Therefore, we emphasize the subtle but important effect of the AES surfactant architecture and its interactions with CNCs to modulate lyotropic LC and Nem mesophase formation.

### CNC in a Solvent-Free State Based on High Excess of Nonionic–Anionic Surfactant: Nematic Thermotropic Liquid Crystalline Behavior

Next, we show that thermotropic liquid crystallinity can be obtained using CNC/AES-mixtures in the absence of an additional organic solvent. We turned to our experience to plasticize molecular-level shape-persistent rod-like π-conjugated polymers poly(aniline) or poly(4-pyridine) to allow thermotropic LC by surfactant complexation. Therein, the surfactants suppress the excessive packing tendency between the macromolecular rods, achieved either by using large surfactant loading or using branched architectures.^50^ Accordingly, herein, we explore whether an excess of nonionic–anionic AES would plasticize CNCs to allow thermotropic LCs even with no addition of organic solvent by colloidal brush architectures. Therefore, we first prepared binary mixtures of CNC/AES^init^ 1:1, 1:5, and 1:10 wt/wt compositions, i.e., low fraction of CNCs and high fraction of the surfactant. We first removed water by freeze-drying, but unlike before, we did not redisperse in toluene or centrifuge, thus leaving the excessive AES for plasticization in the composition. Herein, upon heating above the AES melting temperature, the long alkyl tails of AES play the role of a compatibilizing matrix to CNC/AES complexes. Note that in this case, the final mixture contains the initially administered fraction of AES, and subsequently, we denote the compositions in this section simply as CNC/AES.

First, the thermal stability was investigated. In TGA, the neat AES surfactant shows high thermal stability with a high degradation temperature of ca. 375 °C showing only 2.2 wt % weight loss up to 200 °C, potentially due to the expected small fraction of water, which is almost impossible to remove (Table 1 and Figure S8). Thus, AES acts as a nonvolatile medium/matrix. The thermal degradation temperature for the sodium sulfate form of CNCs is determined to be ca. 254 °C. For the mixtures, the degradation temperature increases with the AES fraction, reaching 370 °C for CNC/AES 1:10 wt/wt. However, we assigned the practical thermal stability regime for the CNC/AES to be 200 °C, above which the material starts to show color changes due to cellulose degradation.

**Table 1:** Solvent-Free CNC/AES Thermal Properties were Determined with TGA and DSC

composition	*T*_degradation_^a^ [°C]	*T*_melting_^b^ [°C]	*T*_crystallization_^c^ [°C]	thermal properties
AES	375	31	–3.5	Cr ⇒ 31 °C Iso
CNC-Na^+^	254	-	–	Cr
CNC/AES 1:5	309	33	–7.0	Cr ⇒ 33°C Nem
CNC/AES 1:10	370	33	–8.3	Cr ⇒ 33°C Nem

a From TGA experiment: 1st degradation peak corresponding to CNCs.

b According to DSC performed with 2.5 °C/min heating–cooling rate.

c Concluded from POM investigations. Cr = crystalline, Nem = Nematic mesophase, and Iso = isotropic.

**Figure 5:**
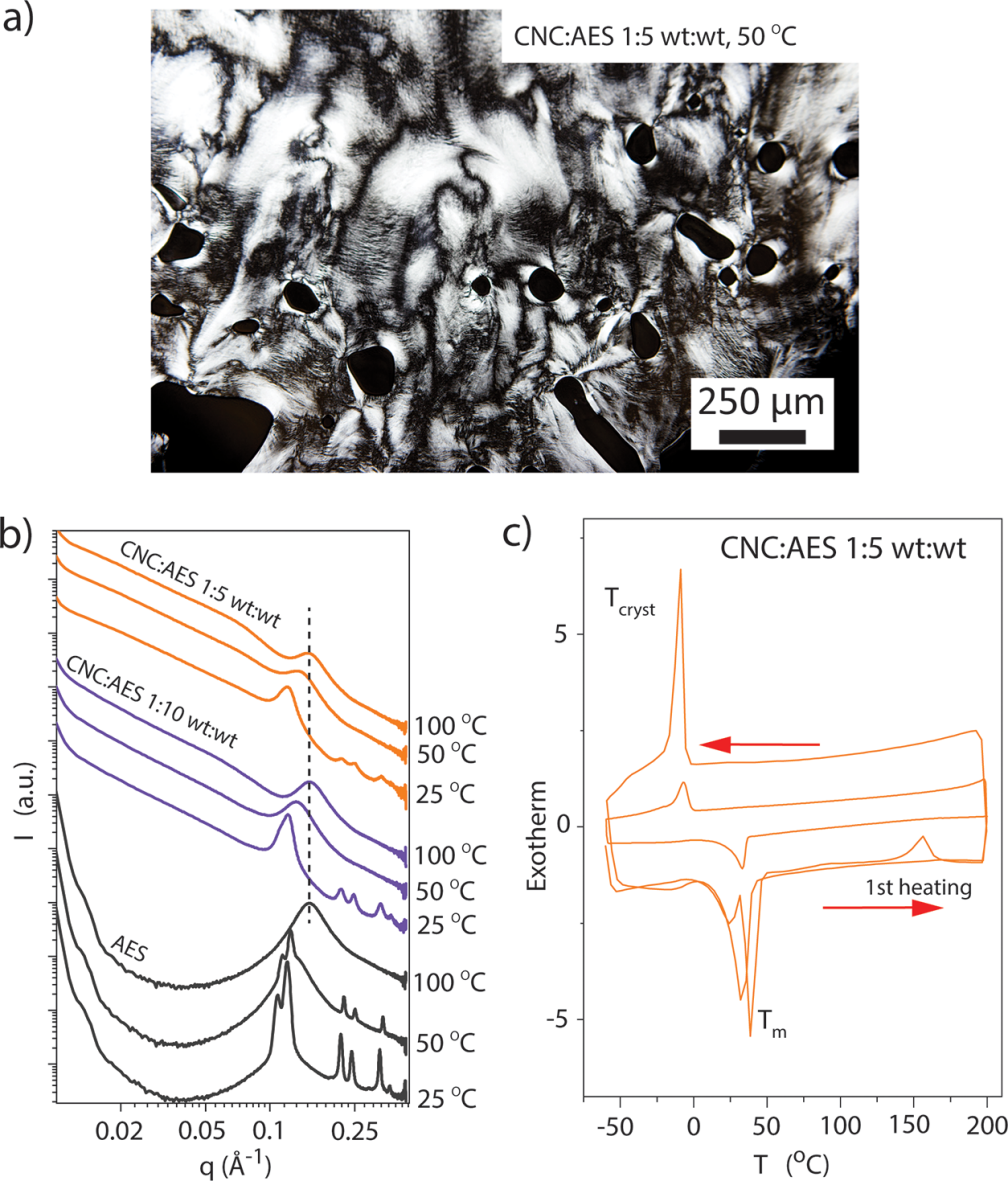
Characterization of the thermotropic LC behavior of CNC/AES compositions at 50 °C. (a) POM microphotograph of CNC/AES 1:5 wt/wt composite, showing a typical Schlieren texture, indicative of a Nem mesophase. (b) 1D SAXS patterns for CNC/AES 1:5 and 1:10 wt/wt, and AES. The SAXS data were acquired during a cooling scan. (c) DSC thermograms for CNC/AES 1:5 wt/wt.

DSC, POM, and SAXS were next used to study the thermal properties and supracolloidal organization. For pure AES, DSC shows a complex history dependence and based on repeated thermal cycles, a melting transition is assigned at 31 °C (Table 1 and Figure S9). CNC/AES 1:1 wt/wt remained solid up to 200 °C, i.e., there was not enough AES in the system to plasticize the CNCs, and such a composition was not explored in more detail. By contrast, CNC/AES 1:5 and 1:10 wt/wt became birefringent fluids upon mild heating. Figure 5a shows a POM microphotograph CNC/AES 1:5 wt/wt at 50 °C demonstrating Schlieren patterns. Also, no fingerprint textures are observed. Therefore, thermotropic Nem LC behavior is inferred, as suggested previously for colloidal rods.^35,36^ CNC/AES 1:5 and 1:10 wt/wt did not show a clearing point below 200 °C, obviously due to the tightly packed colloidal nature of the rod-like mesogens. In SAXS, AES shows several reflections at room temperature but at slightly elevated temperatures, a broad halo is observed at *q* = 0.13 Å^–1^ (AES at 50 °C is undergoing slow crystallization) (see Figure 5b). At 25 °C, traces of reflections of pure AES are observed in CNC/AES 1:5 and 1:10 wt/wt, suggesting phase separation between the components due to crystallization of AES. However, at 50 °C, no reflections of pure AES are observed in the mixtures. Thus, CNC disrupts AES crystallization in the complexes upon heating to provide solvency of the AES, which is a clear indirect sign of the interaction between CNCs and AES (Figure 5b). Thus, the colloidal liquid crystalline nature of the complexes extends the Nem mesophase over a large temperature range.

**Figure 6:**
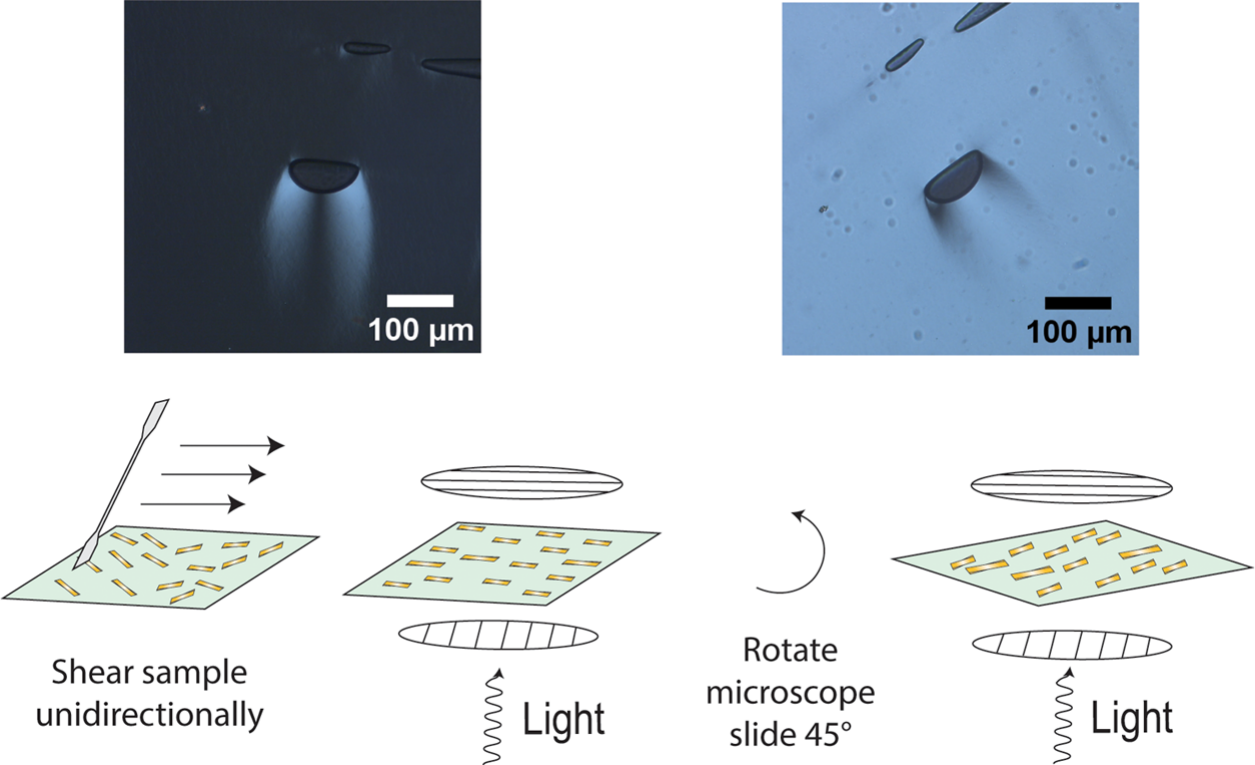
Shear-induced orientation of CNC/AES 1:5 wt/wt thermotropic LC at 50 °C, allowed by its plasticized fluidity. A long-range uniaxial alignment is evident from the difference in the birefringence when the sample director is rotated by an angle of 45° with respect to the crossed polarizers.

Finally, we explored whether the fluid-like thermotropic CNC/AES complexes allow facile shear alignment in the Nem mesophase. Figure 6 shows a schematic illustration that proposes the facile orientation of nanorods in CNC/AES 1:5 wt/wt and the changes upon shearing and rotation of the sample. Under POM, as the sample is turned 45° (hence, the sample director rotating with respect to the crossed polarizers by an angle of 45°) from a state where birefringence is seen only around the air bubble, the imaged area is qualitatively shown to evolve into a highly birefringent one (see also Figure S10 for additional microphotographs of the sheared samples). This demonstrates how the thermotropic LC Nem mesophase of CNC/AES can be shear-oriented. Thus, we propose here that aligning CNC-based thermotropic LC under simple shearing is feasible. Steric effects created by the AES alkyl tail on the surface of the CNC likely hinder the CNC aggregation and lead to thermotropic LC featuring a Nem mesophase. Importantly, the alignment seems stable which is unlike the case with kinetically trapped alignment of chiral Nem* assemblies of CNCs. We confirmed the Nem mesophase with an additional experiment with Video S6 by rotating one polarizer and observing how the Schlieren disclination lines rotate with respect to the disclination point. This can be seen only when rods follow a uniaxial orientation due to shear around a defect point (indicated with a white arrow in the Video S6).

## Conclusions

Herein, we show that a nonionic–anionic trifunctional surfactant (oligo(ethylene glycol) alkyl-3-sulfopropyl diether potassium salt, i.e., alcohol ethoxy sulfonate AES), suppresses the typically observed inherent cholesteric LC of CNCs, leading to nematic lyotropic LC in toluene and nematic thermotropic LC in excess surfactant, i.e., in the absence of the solvent. We show the binding of AES on CNC in toluene by D-QCM and FTIR spectroscopy, indicating hydrogen bonding between the oligo(ethylene glycol) block of AES and CNC hydroxyl groups, suggesting a dense adsorbed surfactant layer with the hydrophobic tails exposed, allowing low viscosity supracolloidal brush architecture in toluene. Control experiments with the corresponding nonionic ethoxylated alcohol surfactant, i.e., in the absence of the potassium sulfonate terminal group of AES, did not allow dispersibility in toluene, LC, or nematic behavior. This pinpoints the essential role of a dense set of charges and electrostatics even in the nonaqueous phase, allowed by AES in nonaqueous media. We have identified the key-enabling structural features a surfactant should possess (CNC binding block, hydrophobic block, and charged block) to form an organophilic lyotropic liquid crystal with a Nem mesophase, opening doors to the identification of alternative surfactant candidates. Importantly, using a plasticizing AES also leads to a neat, i.e., in the absence of an additional solvent, CNC/AES thermotropic liquid crystal showing a nematic mesophase upon heating, providing that the amount of the surfactant is sufficiently large. It can be easily aligned by mild shearing and heating. The nematic alignment of CNCs made possible by surfactants such as AES is expected to open new opportunities that benefit from highly aligned assemblies including nanocomposites, filament reinforcement, and polarizers.

## Supporting Information Available

Evaluation of the surfactant crowding, size-distribution characterization, NMR characterizations, additional POM characterizations, demonstration on the thixotropic behavior, DLS results, TGA and DSC thermograms (PDF)
Rotation of Schlieren brushes in CNC/AES complexes at 16 wt % concentration in toluene, when both crossed polarizers are rotated (Video S1) (MP4)
Fluidity of sample upon pressing of the cover glass, followed by rotation of Schlieren texture in CNC/AES (16 wt % in toluene) when both crossed polarizers are rotated (Video S2) (MP4)
Fluidity of sample upon pressing of the cover glass and formation of new Schlieren texture in CNC/AES (16 wt % in toluene) (Video S3) (MP4)
CNC/AES complex dispersion in toluene at 16 wt % concentration show pronounced flow birefringence upon shearing. Imaged with sample between crossed polarizer films (Video S4) (MP4)
Fluid like CNC/AES complex dispersion in toluene at 16 wt % concentration. Imaged with sample between crossed polarizer films (Video S5) (MP4)
Rotation of a Schlieren disclination line for a CNC/AES 1:5 wt/wt composite at 150 °C under POM, when one of the polarizer is rotated (Video S6) (MP4)
