# Supplementary material for: Controlling the Nematic Liquid Crystallinity of Cellulose Nanocrystals with an Alcohol Ethoxy Sulfonate Surfactant
Source: Biomacromolecules. 2024 Mar 20;25(7):3909–19. doi: 10.1021/acs.biomac.3c01375 (PMC11238325; doi:10.1021/acs.biomac.3c01375)
Supplement: Supplementary file 1 — bm3c01375_si_001.pdf [file bm3c01375_si_001.pdf]

Supporting information to:

Controlling nematic liquid crystallinity of cellulose nanocrystals with alcohol ethoxy sulfonate surfactant

Johanna Majoinen[a,b‡], Lotta Gustavsson[c‡], Owies Wani[c], Samira Kiefer[c], Ville Liljeström[d], Orlando J. Rojas[a,e], Patrice Rannou[f]* and Olli Ikkala[c]**

[a] Department of Bioproducts and Biosystems, Aalto University, FIN-00076 Aalto, Espoo, Finland

[b] Technical Research Centre of Finland VTT, Biomaterial Processing and Products, FI-02150, Espoo, Finland

[c] Department of Applied Physics, Aalto University, Espoo FI-00076, Finland

[d] Nanomicroscopy Center, OtaNano, Aalto University, FI-00076, Espoo, Finland

[e] Bioproducts Institute, Department of Chemical and Biological Engineering, Department of Chemistry and Department of Wood Science, University of British Columbia, 2360 East Mall, Vancouver, BC Canada V6T 1Z3

[f] Université Grenoble Alpes, Université Savoie Mont-Blanc, CNRS, Grenoble INP, LEPMI, 38000 Grenoble, France

‡ Equal contribution to the article

*Correspondence to patrice.rannou@grenoble-inp.fr, owies.wani@aalto.fi, olli.ikkala@aalto.fi

Evaluation of the surfactant crowding of AES on the CNC surfaces based on centrifugation

Suppose:

d = CNC lateral width = 11 nm

L = CNC length

ρ= CNC density = 1.5 g/cm^3

M_w= AES molecular weight determined with NMR = 770 g/mol

A = Avogadro number

Surface area of one CNC: $S = 4\,dL$

Weight of CNC = $\rho d^{\,2}L$

The observed bound weight fraction of AES corresponds to CNC:AES 1:0.99 w:w ≈ 1:1 determined by weighting the sample after removal of unbound AES.

Therefore, the weight of bound AES = $\rho d^{\,2}L$

Number of moles of bound AES = $\rho d^{\,2}L/M_w$

Number of bound AES molecules = $\rho d^{\,2}LA/M_w$

Number of bound AES molecules/CNC surface area = $(\rho d^{\,2}LA/M_w)/S = \rho dA/(4M_w)$ = 3.2 AES/(nm)^2, indicating a highly crowded surface for a CNC.

Evaluation of the surfactant crowding of AES on the CNC surfaces based on QCM-D

Sauerbrey equation: 0.65 mg/m^2

For 1 m^2: AES: 0.65 10^{-3} g -> 0.65 10^{-3}/770 moles -> (0.65 10^{-3}/770) x 6.02 10^{23} molecules -> 0.5 AES/(nm)^2

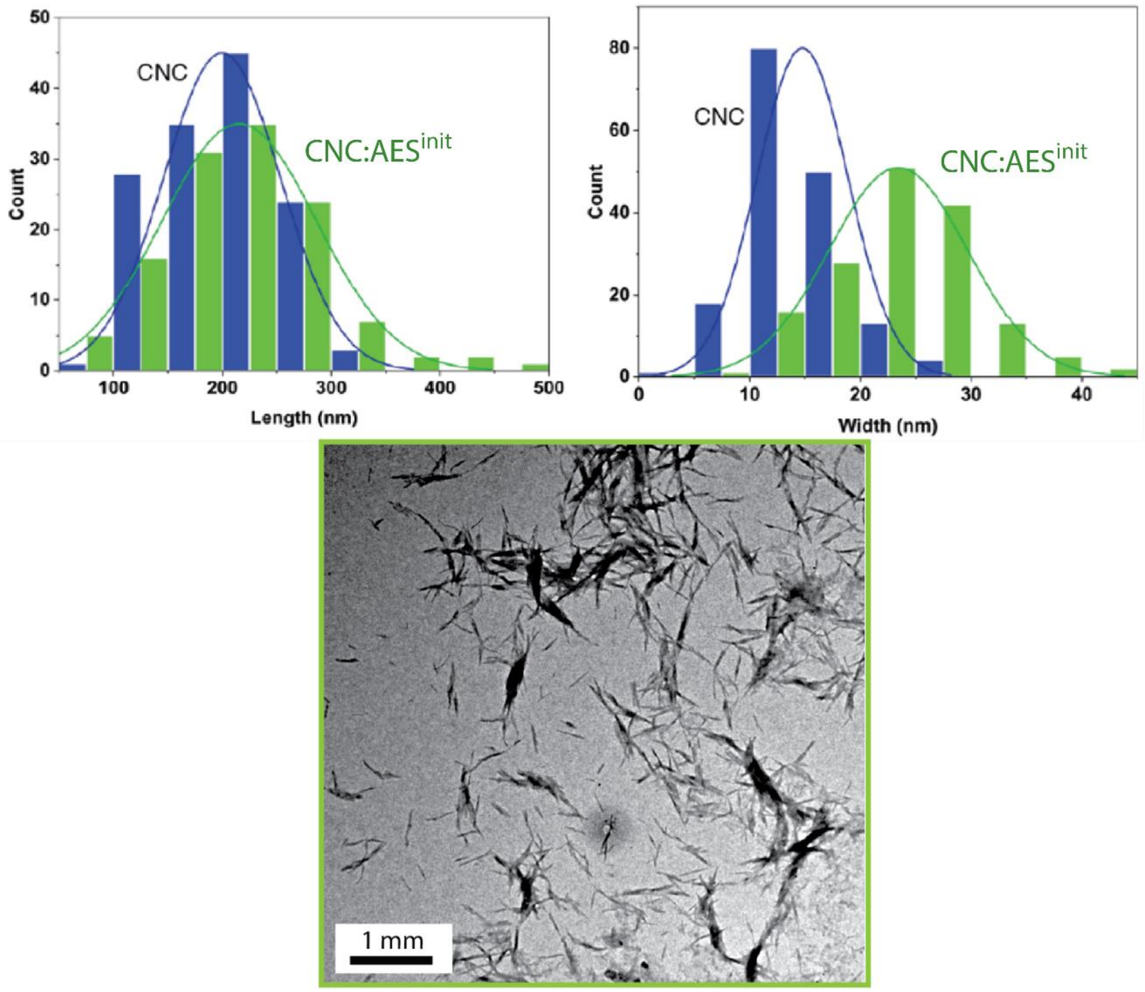

Figure S1. The size (length and width) distributions based on TEM analyses of pristine CNCs dispersed in water and CNC:AES^{init} complexes (depicted in TEM image) dispersed in toluene.

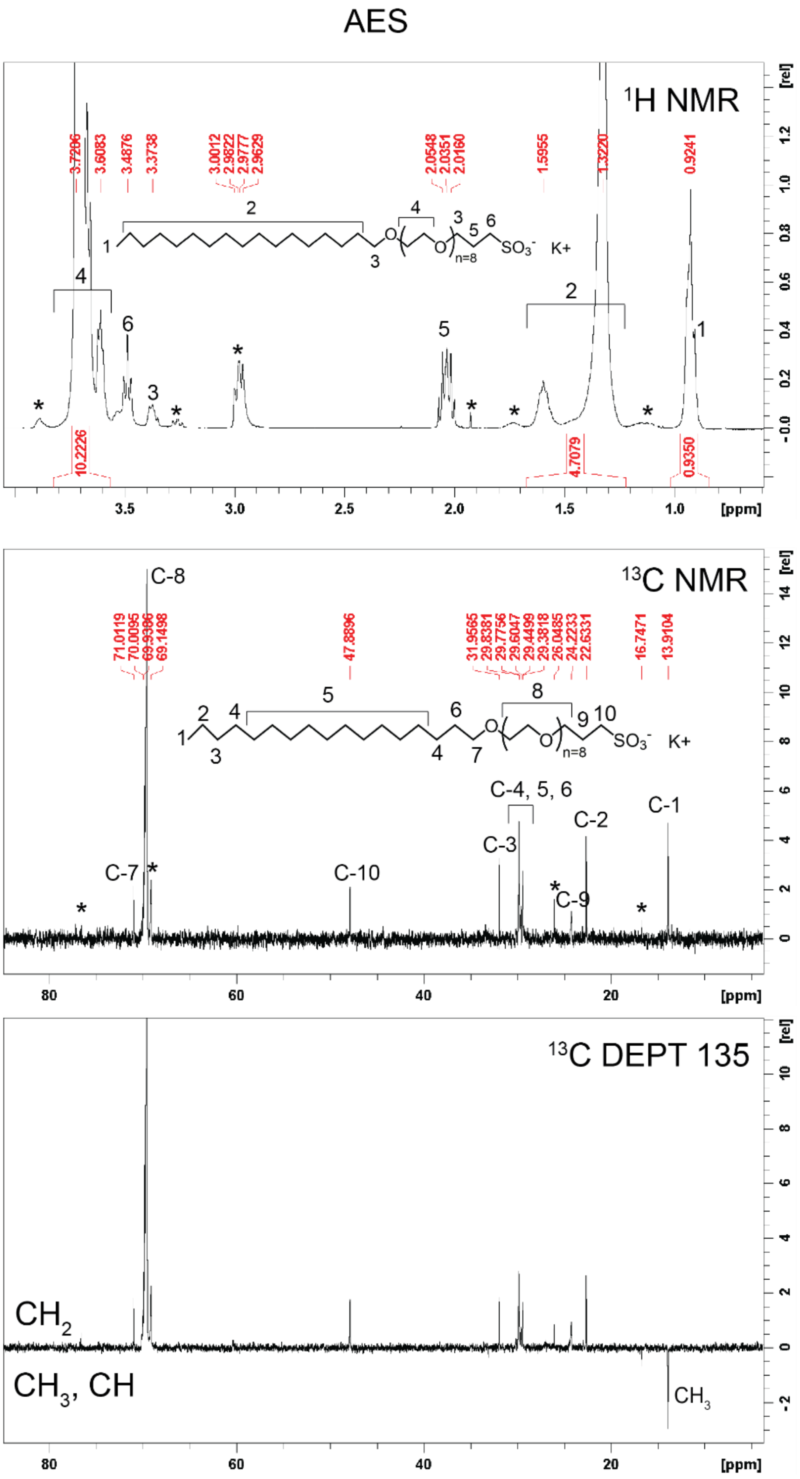

Figure S2. ^{1}H, ^{13}C, and ^{13}C DEPT-135 NMR analyses of surfactant AES. The ^{1}H NMR spectrum is calibrated with corresponding D_2O signal. Unassigned peaks are marked with *.

AE

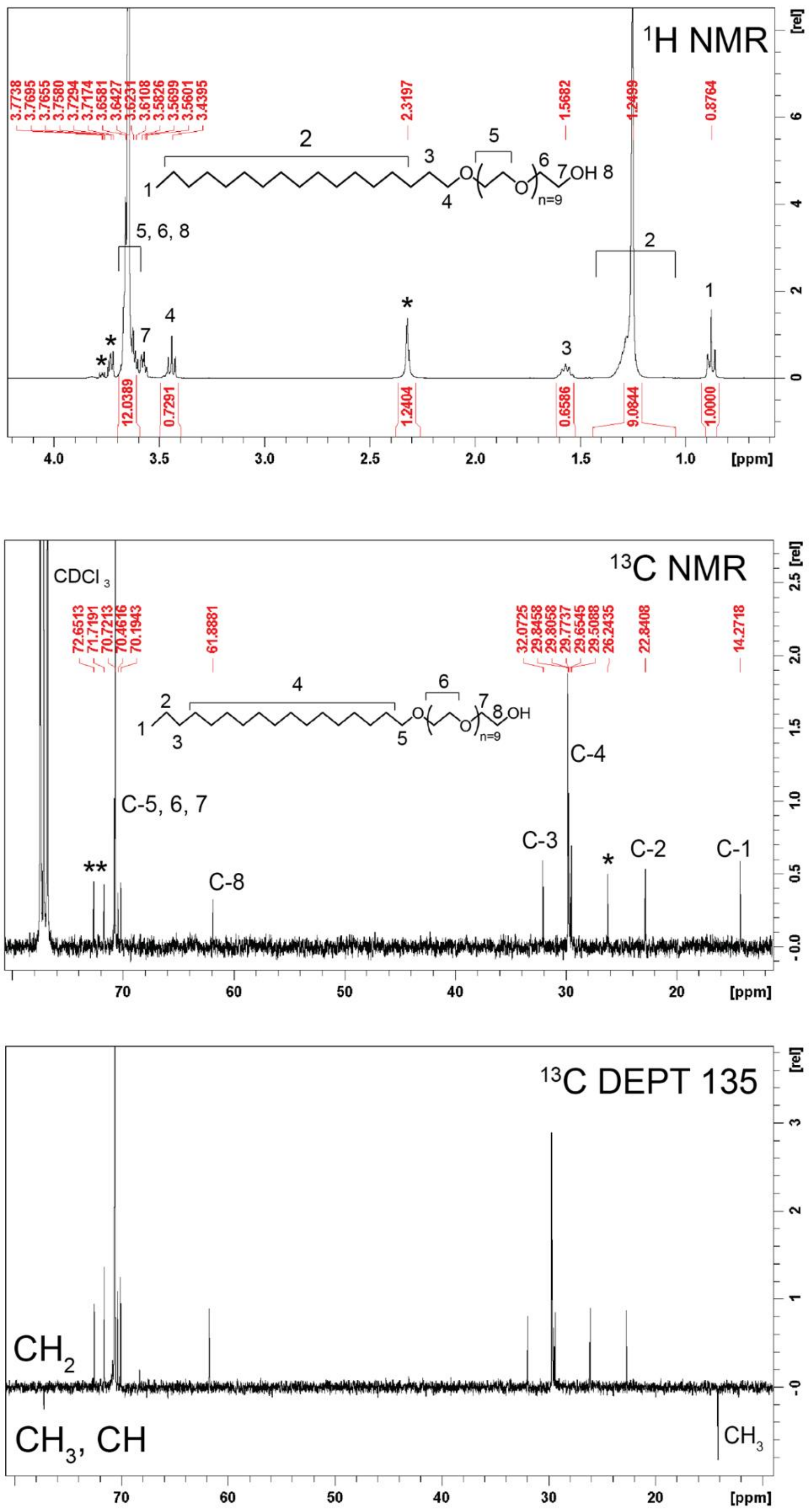

Figure S3. ^{1}H, ^{13}C, and ^{13}C DEPT-135 NMR analysis of surfactant AE. ^{1}H and ^{13}C NMR spectra are calibrated with corresponding $CDCl_3$ signal. Unassigned peaks are marked with *.

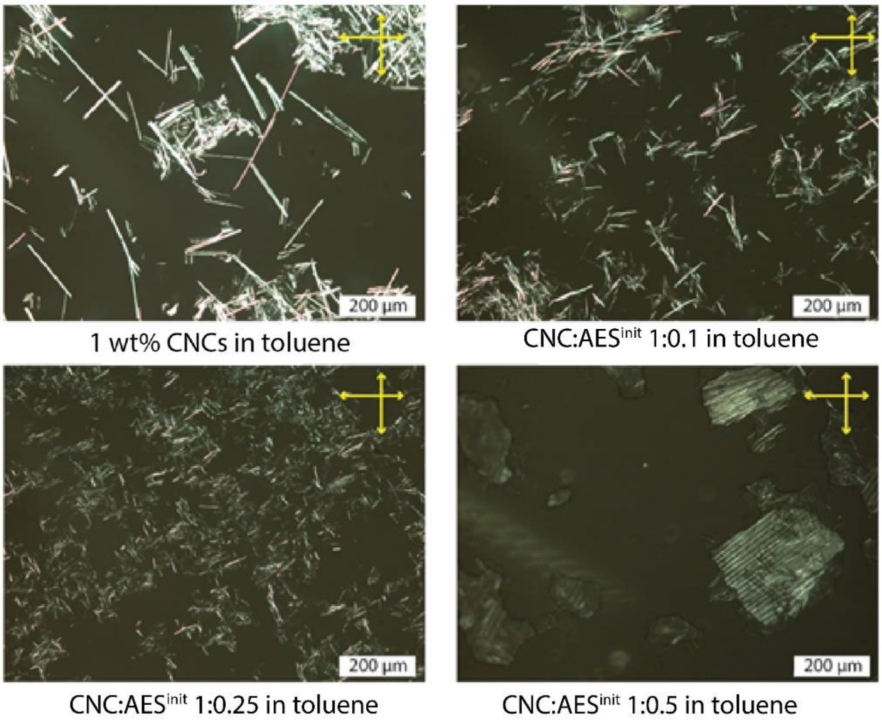

Figure S4. POM microphotographs of CNCs and CNC:AES^{init} compositions with their different weight fractions in toluene before any washing steps to remove the unbound AES. Pure CNCs and CNC:AES^{init} at very low AES fraction (1:0.1 wt:wt) form needle-like crystallites. Upon increasing the AES fraction vs. CNCs, softer boundaries become evident, until at 1:0.5 wt:wt soft aggregates with striped patterns are observed, resembling chiral nematic packing of the components. Yellow arrows indicate the crossed linear polarizer directions.

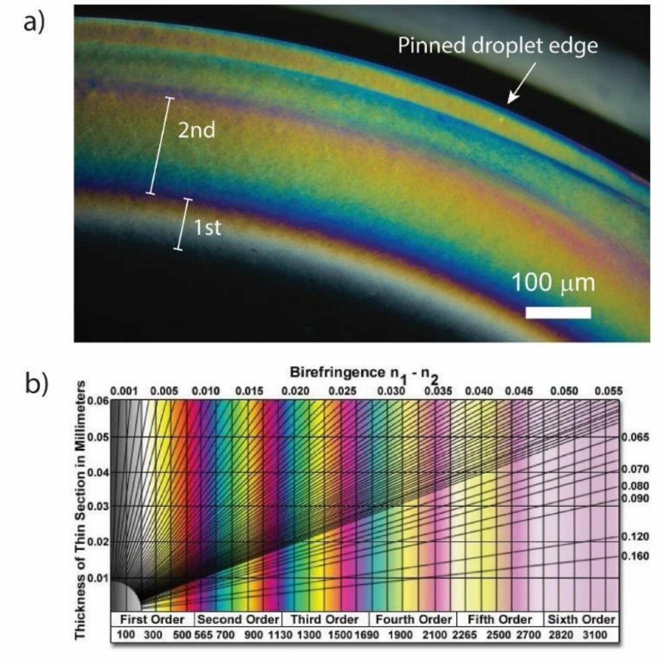

Figure S5. a) POM microphotograph of a 16 wt% CNC:AES complex dispersion in toluene between glass slides. The CNC:AES complexes drift and stack at the pinned droplet edge. A high degree of nematic alignment is evident with the appearance of interference coloring and increasing birefringence according to b) Michel-Lévy chart.[S1]

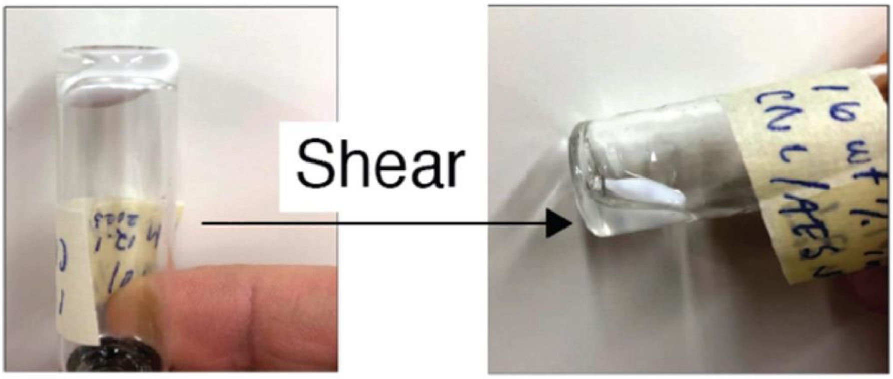

Figure S6. CNC:AES 16 wt % toluene suspension showing a thixotropic behavior a) The toluene-based gel is jammed in the vial after aging for 24 h to form a soft gel. But when stirred, it regains easily its fluid-like behaviour .

Table S1. DLS results from CNCs, AES surfactant, and CNC:AES complexes in toluene and in water. All values are averaged values from three measurements. Size values reported are z-average values.

Sample	Zeta [mV] / Size [nm] in toluene	Zeta [mV] / Size [nm] in water
CNC [a]	---	-45 / 136
AES [b]	--- (data inconclusive)	-22 / 276
CNC:AES [c]	1 / 568	-31 / 79

[a] concentration 0.5 mg/mL. [b] concentration 100 mg/mL [c] concentration 5.8 mg/mL.

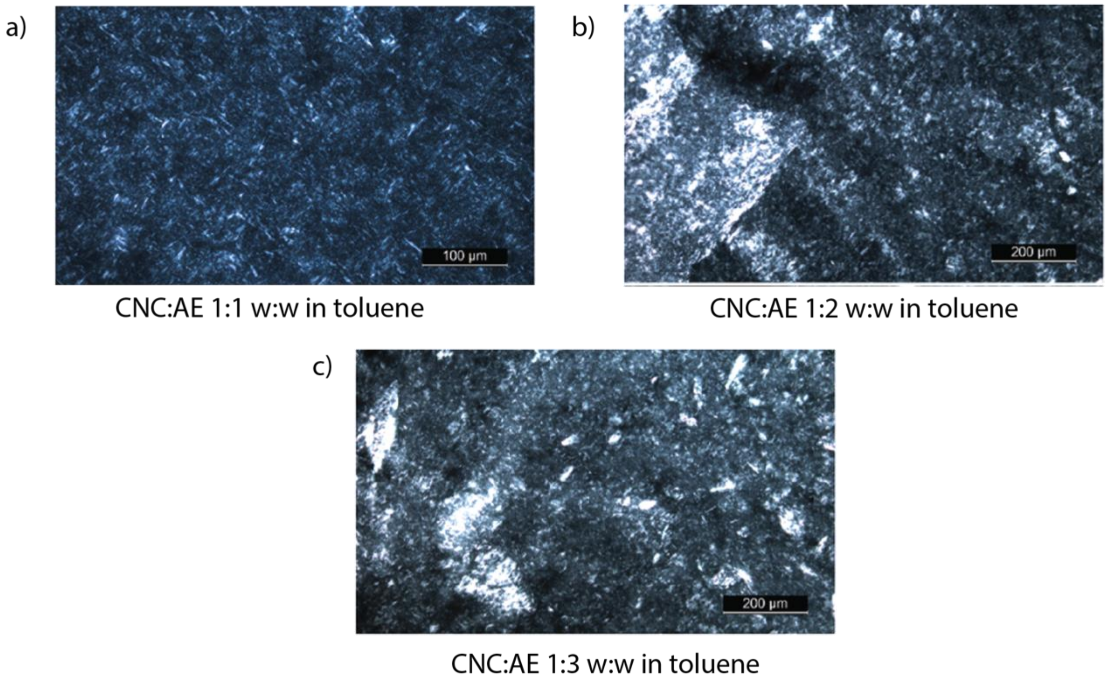

Figure S7. POM microphotographs of CNC:AE^{init} complexes in toluene using the reference surfactant AE (2.5 wt% CNC) with different compositions. Aggregates can be seen floating in toluene, without any indication of lyotropic LC phase formation.

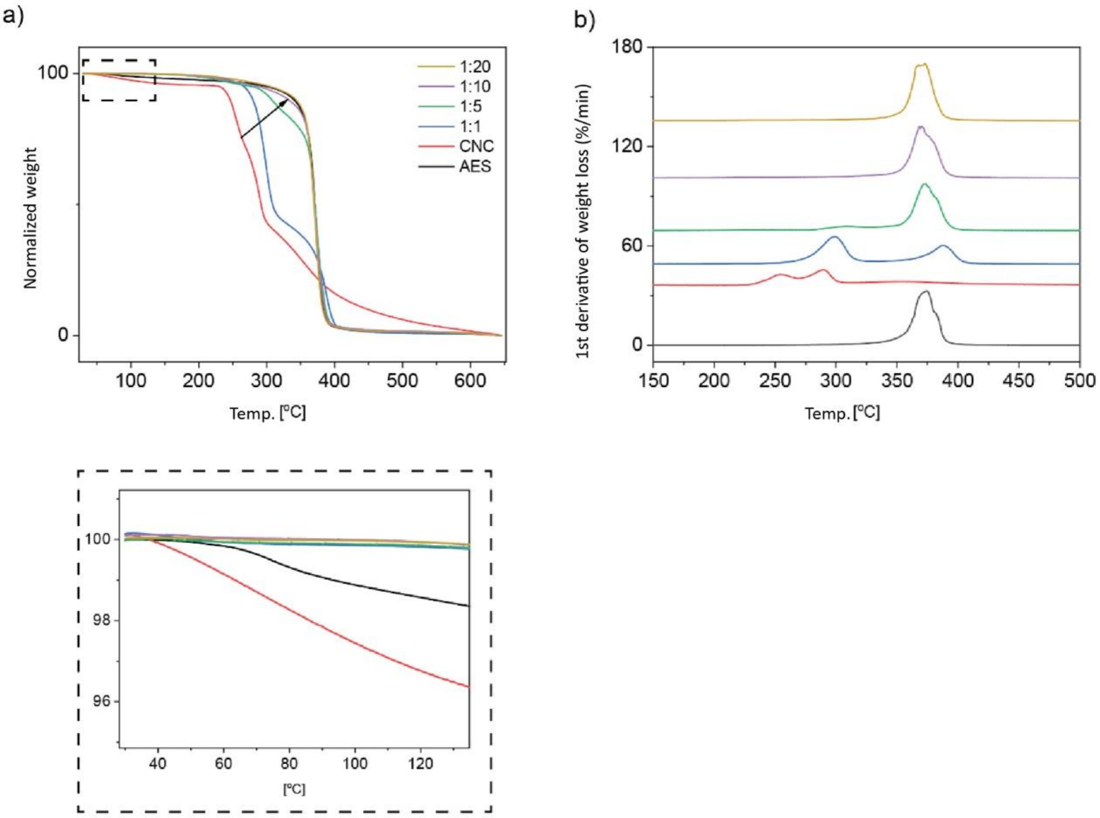

Figure S8. Thermogravimetric analysis (TGA) results for pure components AES and CNCs and their composites with weight ratios wt:wt 1:1, 1:5, 1:10 and 1:20. a) Normalized weight versus temperature including a zoomed-in section for the low-temperature region. While the components CNC and AES alone show water loss, the freeze-drying efficiently removes the bound water and the composites do not show any detectable mass loss, indicating a dry complex. b) The 1st derivative of weight loss versus temperature. The complexes with more AES (1:5, 1:10, and 1:20 wt:wt) do not show the thermal decomposition of the pure CNC. This suggests that the AES surfactant protects the CNC from thermal decomposition when the coverage is sufficient.

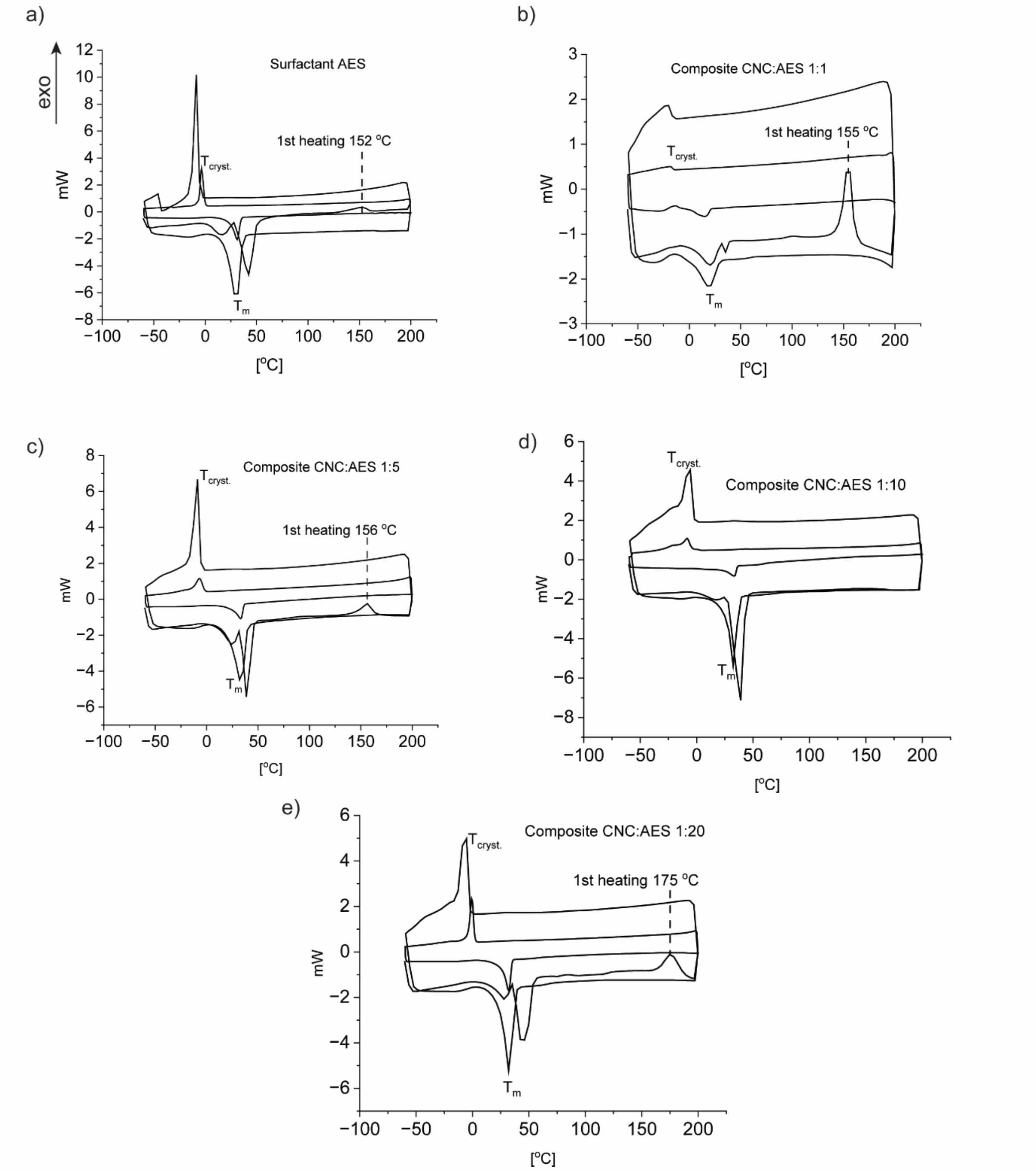

Figure S9. Differential scanning calorimetry (DSC) thermograms for a) pure AES; as well as for the CNC:AES (wt:wt) composites b) 1:1, c) 1:5, d) 1:10 and e) 1:20. The melting and crystallization peaks of the complex 1:1 wt:wt are small, and in line with the POM investigations where no bulk melting is observed for the sample. The thermal cycles were measured with the following scanning rates: 1st heating-cooling-heating cycle: 10 °C/min and 2nd cooling-heating cycle: 2.5 °C/min. Upon heating at 10 °C/min, cold crystallization is seen with every sample before melting.

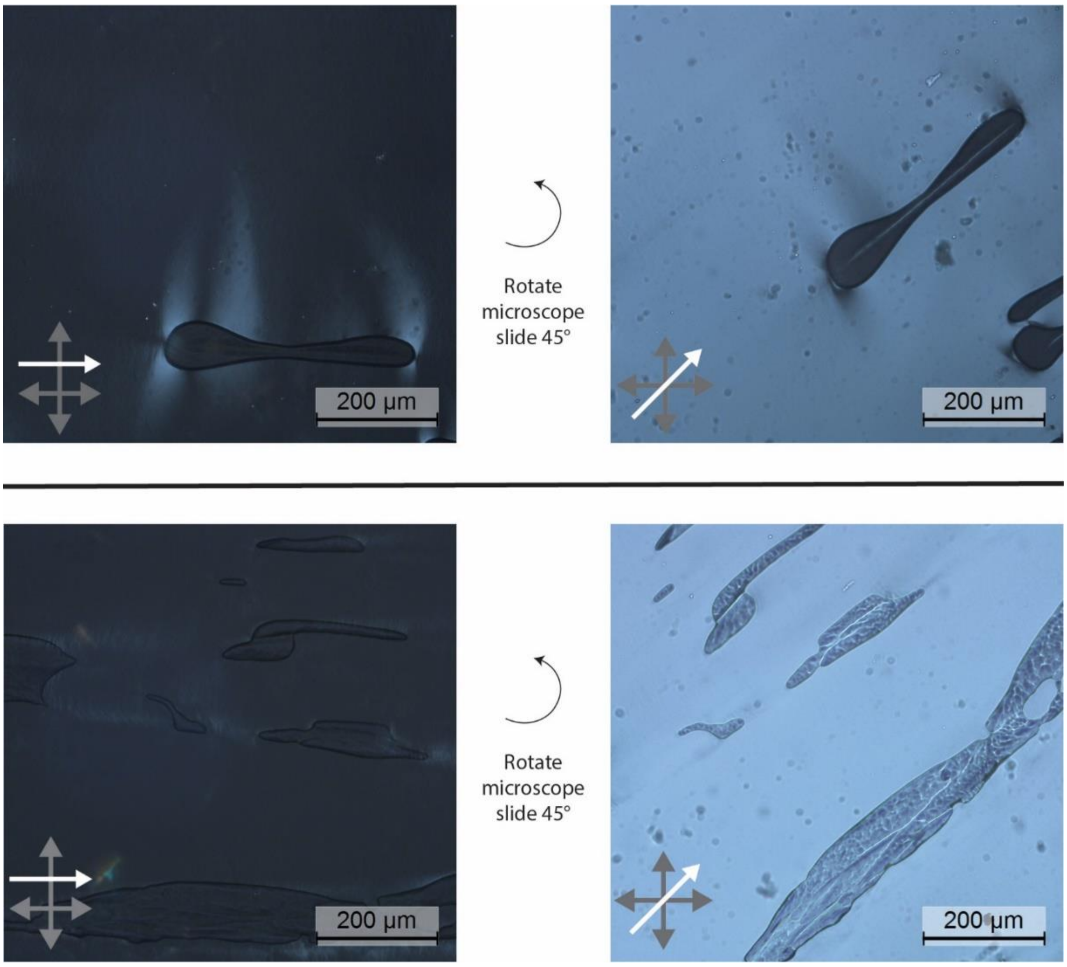

Figure S10. Additional POM microphotographs of CNC:AES 1:5 wt:wt at 50 °C. The gray arrows represent the polarizing filters and the white arrow the direction of shearing with respect to the crossed polarizers.

Supporting reference

[S1] Sørensen, B.E. A revised Michel-Lévy interference colour chart based on first-principles calculations, *Eur. J. Mineral.* **2013**, 25(1), 5–10. https://doi.org/10.1127/0935-1221/2013/0025-2252 .

Supporting Videos

Video S1. Video depicts rotation of Schlieren brushes in CNC:AES complexes at 16 wt % concentration in toluene, when both crossed polarizers are rotated.

Video S2. Video depicts fluidity of sample upon pressing of the cover glass, followed by rotation of Schlieren texture in CNC:AES (16 wt % in toluene) when both crossed polarizers are rotated.

Video S3. Video depicts fluidity of sample upon pressing of the cover glass and formation of new Schlieren texture in CNC:AES (16 wt % in toluene).

Video S4. CNC:AES complex dispersion in toluene at 16 wt % concentration show pronounced flow birefringence upon shearing. Imaged with sample between crossed polarizer films.

Video S5. Fluid like CNC:AES complex dispersion in toluene at 16 wt % concentration. Imaged with sample between crossed polarizer films.

Video S6. Video depicts the rotation of a Schlieren disclination line for a CNC:AES 1:5 wt:wt composite at 150 °C under POM, when one of the polarizer is rotated.
